# Evolutionarily conserved coding properties favour the neuronal representation of heterospecific signals of a sympatric katydid species

**DOI:** 10.1007/s00359-018-1282-0

**Published:** 2018-09-17

**Authors:** Konstantinos Kostarakos, Heiner Römer

**Affiliations:** Institute of Biology, University of Graz, Universitaetsplatz 2, 8010 Graz, Austria

**Keywords:** Insects, Auditory processing, Signal detection, Signal evolution, Selective coding

## Abstract

To function as a mechanism in premating isolation, the divergent and species-specific calling songs of acoustic insects must be reliably processed by the afferent auditory pathway of receivers. Here, we analysed the responses of interneurons in a katydid species that uses long-lasting acoustic trills and compared these with previously reported data for homologous interneurons of a sympatric species that uses short chirps as acoustic signals. Some interneurons of the trilling species respond exclusively to the heterospecific chirp due to selective, low-frequency tuning and “novelty detection”. These properties have been considered as evolutionary adaptations in the sensory system of the chirper, which allow it to detect signals effectively during the simultaneous calling of the sympatric sibling species. We propose that these two mechanisms, shared by the interneurons of both species, did not evolve in the chirper to guarantee its ability to detect the chirp under masking conditions. Instead we suggest that chirpers evolved an additional, 2-kHz component in their song and exploited pre-existing neuronal properties for detecting their song under masking noise. The failure of some interneurons to respond to the conspecific song in trillers does not prevent intraspecific communication, as other interneurons respond to the trill.

## Introduction

Given the advantages of long-range signalling by airborne sound, it is not surprising that some insect taxa, such as grasshoppers, crickets, katydids, cicadas and even some groups of moths, have evolved airborne sound signals for acoustic communication (von Helversen and von Helversen 1994; Gerhardt and Huber 2002; Greenfield 2002). These signals are usually produced by males, and their function has been thoroughly documented in contexts of male competition, spacing, courtship and mate choice (Gerhardt and Huber 2002; Bradbury and Vehrenkamp 2011; Pollack et al. 2016). The songs produced show great diversity in their temporal patterns even in closely related species, and therefore, have received much attention as a potential premating isolation mechanism. Whatever the primary reason for this diversity may have been (adaptation to changes in the physical and biotic environment, natural or sexual selection, or genetic drift; Panhuis et al. (2001), Safran et al. (2013), Wilkins et al. (2013)), modifications in acoustic signal parameters that result in divergence between populations can lead to speciation (West-Eberhard 1983; Coyne 1992).

The diversity of these signals is achieved by the modification of a single sound pulse as the basic unit into a continuous series of pulses (trills) or discontinuous, short sequences of pulses (chirps) with species-specific pulse intervals and durations, which are repeated within a specific chirp period. Depending on the physical properties of the sound generators, these temporal patterns are broadcast as pure-tone or broad-band signals. Thus, small changes in the neuronal pattern generators for the leg or wing movements during sound production can create modifications in the song pattern. The females can subsequently use these modifications as a basis for discrimination and avoid being attracted towards heterospecific mates (Elsner 1983). This view is supported by the results of genetic analysis of quantitative trait loci in *Drosophila* and an acoustic moth, which suggest that an allele change at a single locus could result in the production of a markedly different signal (Gleason et al. 2002; Limousin et al. 2012).

To function as a premating isolation mechanism, female receivers should be selective for the song pattern produced by males of their own species. This has been confirmed in many studies using no-choice or two-choice behavioural paradigms in grasshoppers, katydids and crickets (Popov and Shuvalov 1977; Doherty 1985; Hoy 1990; Henry 1994; von Helversen and von Helversen 1994; Schul et al. 1998; Shaw and Herlihy 2000; Gerhardt and Huber 2002; Blankers et al. 2015; Hennig et al. 2016; Bailey et al. 2017, but see Doherty and Howard (1996) for a negative result). For example, Hennig et al. (2016) examined male signal and female preference co-variation in three closely related, allopatric species of *Gryllus* crickets and quantified the male song traits as well as multivariate female preferences. They showed that males differed strongly in either the song carrier frequencies or pulse rates. The female preference functions for these traits also differed and, in combination, allowed for species discrimination. In contrast, the dynamic song traits, such as chirp rate and chirp duty cycle, showed minimal divergence among species and relatively greater conservation of female preference functions.

The correct interpretation of acoustic signals is based on the processing properties of the auditory pathway of receivers. Given the behavioural specificity reported above, we would expect that filters exist in the frequency and time domain at some stage during processing that enable the female to respond only to a conspecific male’s song. Elements of such filters have been described in grasshoppers, katydids and crickets (Wehner 1987; Ronacher and Stumpner 1988; Hedwig and Pollack 2008; Kostarakos et al. 2009; Kostarakos and Römer 2015; Schöneich et al. 2015; Römer 2016). Research conducted on one of the best-studied model systems (field crickets) allowed the identification of a small network of brain nerve cells. The responses of these cells to acoustic signal variants in the time domain exactly match the behavioural selectivity of female phonotactic responses (Schildberger 1985; Kostarakos and Hedwig 2012; Schöneich et al. 2015).

To demonstrate how the ear, the afferent auditory pathway and the brain have been shaped by selection pressure(s) that has led to the selective coding of acoustic signals, the most straightforward approach would be to directly compare homologous units of the auditory pathway in two closely related species with divergent songs. Taking such an approach would reveal the necessary adaptations in neuronal properties that result in species-specific responses. Surprisingly, however, we are aware of only three studies in which such approach was taken. Neuhofer et al. (2008) addressed the question of whether the auditory pathway of acridid grasshoppers may have responded to selection pressures by developing specific adaptations for the processing of communication signals. They compared the properties of identified auditory neurons in two grasshopper species that are not closely related, namely, *Chorthippus biguttulus* (Gomphocerinae) and *Locusta migratoria* (Oedipodinae). These two taxa have a long history of separation (Early Tertiary or even Cretaceous period; Flook and Rowell 1997). Moreover, sound signals only play an important role as a prime cue for mate finding and mate choice in *Ch. biguttulus*, but not in the locust. Using the species-specific communication signals of *Ch. biguttulus* and comparing spike trains in responses of homologous neurons, Neuhofer et al. (2008) found no significant differences between both species. This high degree of correspondence indicates that the coding properties were strongly evolutionarily conserved.

**Fig. 1:**
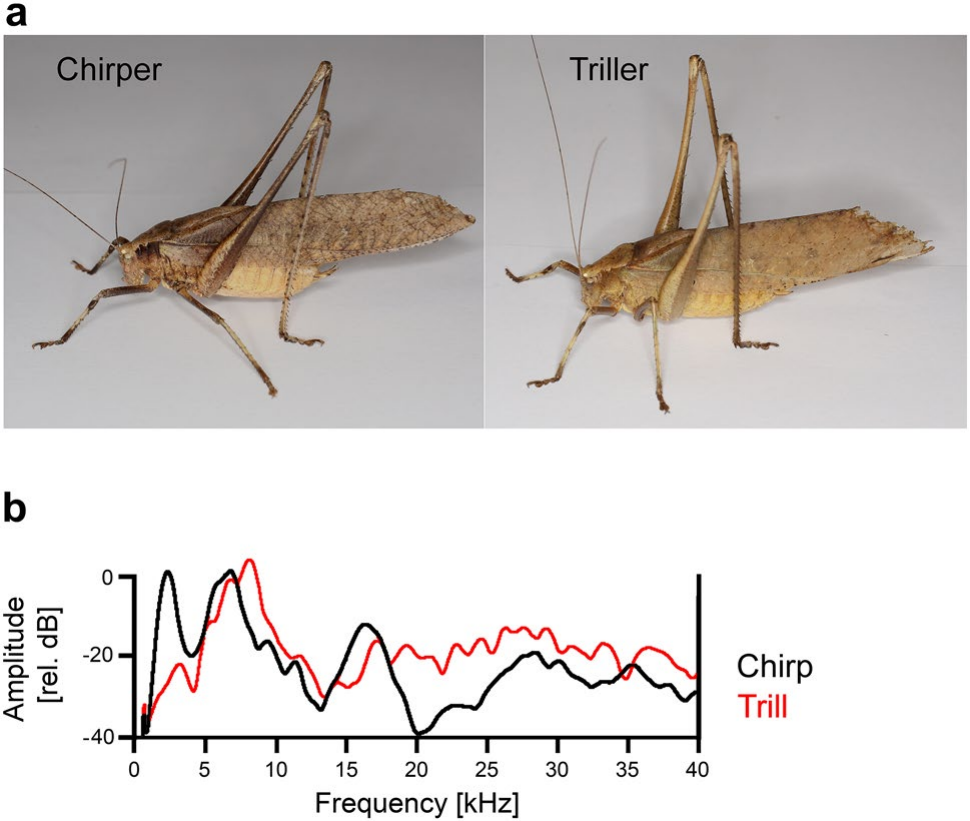
**a** Photographs of the “triller” and “chirper” (courtesy Wolfgang Gessl, Graz). **b** Spectograms of the calling songs of chirpers (black) and trillers (red) recorded at the position of the preparation. Note the higher amplitude at 2 kHz in the chirper spectrum

However, two other studies (Stumpner 2002; Triblehorn and Schul 2009) compared some homologous interneurons in related species of katydids with respect to their spectral tuning or temporal processing, and the authors came to a different conclusion. In the present study, therefore, we conducted a comparison between two sympatric, sibling species of katydids. The two species of the *Mecopoda elongata* complex (Ensifera, Tettigoniidae, Mecopodini) communicate acoustically with extremely different calling songs. Males of the one species produce chirps with regular inter-chirp intervals of about 2 s (Hartbauer et al. 2012; Siegert et al. 2013); they are identical to those of “species S” described by Sismondo (1990). The song of the other species consists of an amplitude-modulated song motif, followed by a long-lasting trill (Krobath et al. 2017). In the following text, both species are referred to as “chirper” and “triller” (see photographs in Fig. 1a). Songs of the triller are identical to those of “*Mecopoda* sp. 2” (Korsunovskaya 2008). In the same genus *Mecopoda*, five other song types have been described in Southern India (Nityananda and Balakrishnan 2006; Dutta 2015). Four of the species with these song types were morphologically indistinguishable, but their songs differed greatly, ranging from simple, short chirps to highly complex songs with multiple components. Species are sympatric and have synchronous breeding seasons.

Given that the spectra of the songs of the chirper and triller strongly overlap, and the continuous song of triller is much louder than the song of the chirper (103 dB SPL at 15 cm distance), it was surprising that a behavioural detection threshold at a signal-to-noise ratio (SNR) of − 9 dB was observed for the chirps under masking conditions of the triller song (Siegert et al. 2013). The aim of a previous study was, therefore, to determine the particular coding properties of receptors and interneurons in the afferent auditory pathway of the chirper that allow the representation of the conspecific chirp, despite the presence of strong masking sounds produced by the sympatric species (Kostarakos and Römer 2015). We demonstrated that chirpers use two basic neural mechanisms for signal identification under these masking conditions. (1) Selective tuning: Auditory interneurons respond selectively to the chirp, as they are sharply tuned to a low-frequency component around 2 kHz, which has a high level of sound energy in the calling song of chirpers, but a reduced amplitude in the calling song of trillers. These neurons were observed to respond selectively to the chirp, as they were unaffected by the higher frequencies of the trill. (2) Novelty detection: Auditory interneurons are broadly tuned, including the frequency spectrum of the trill, but they respond selectively to the repetitive chirp presented at the same time due to strong degree of adaptation to the continuous trill, as the low-frequency component in the chirp represents a “novelty” (Schul et al. 2012; Schul and Sheridan 2006). These two mechanisms sufficiently explain the fact that the chirp is coded reliably even at low signal-to-noise ratios. The mechanisms were proposed to be evolutionary adaptations of the afferent auditory pathway, which allow signals to be detected under the strong masking conditions imposed by the sympatric sibling *Mecopoda* species. Alternatively, we suggested that these mechanisms represent pre-existing properties of a common ancestor (Kostarakos and Römer 2015).

Based on these findings, the obvious question arises which specific adaptations exist in the response properties of homologous interneurons in the auditory pathway of the triller, favouring the representation of the trill. The two mechanisms found in the chirper, namely, selective sensitivity for the 2 kHz component and novelty detection, favour the representation of the heterospecific rather than conspecific song. A comparative analysis of the response properties of homologous auditory interneurons in both species has great potential to reveal how response properties of neurons have been shaped by environmental constraints and/or sexual selection. In the present study, therefore, we analyzed how auditory interneurons of the triller responded to conspecific and heterospecific songs under masking and unmasked conditions. The data collected were compared to those presented for the chirper species in Kostarakos and Römer (2015), allowing us to draw conclusions regarding the evolution of neural features in the auditory system of receivers.

## Materials and methods

### Animals

Insects were raised in a colony at the University of Graz. Individuals of the triller species were originally collected in Malaysia and used to establish this colony. The insects were reared in a 12-h light/dark cycle at a temperature of 27 °C and 70% relative humidity. They were fed on fresh lettuce, oat flakes and fish food ad libitum.

### Acoustic stimulation

To investigate the potential species-specific coding properties of interneurons in the triller, we used the calling songs of both the chirper and the triller for acoustic stimulation. The songs were recorded from isolated males at a distance of 15 cm from the signalling individual, using a calibrated free-field condenser microphone (type 40AC, G.R.A.S. Sound & Vibration A/S, Holte, Denmark) with a flat frequency response between 10 Hz and 40 kHz. The microphone output was amplified using a preamplifier (type 26AM, G.R.A.S. Sound & Vibration A/S) and a power module (type 12AK, G.R.A.S. Sound & Vibration A/S). A/D conversion was performed via an external audio interface (Edirol FA-101, Roland Inc., Tokyo, Japan), operating at a sampling rate of 96 kHz.

The chirp used for playback consisted of 15 syllables (chirp duration 285 ms, syllable period 20 ms) with a gradually increasing amplitude and was repeated at a rate of 0.4 Hz. The song of the triller was more complex, with two motifs: an amplitude-modulated (AM-) motif and a trill. These motifs differed mainly in their amplitude, but also in the fine structure of the syllables. While the AM-motif consisted of alternating loud and soft segments, the trill had a continuously high amplitude of 103 dB SPL when the signalling individual was 15 cm from the male, in contrast to 86 dB SPL for soft segments (Krobath et al. 2017). Both the loud song segments in the AM-motif and the trill consisted of a soft syllable followed by two syllables with high amplitude, a stereotypical pattern that was repeated with a period duration of 30 ms for more than 100 s. We used this trill pattern as conspecific stimulus in our playbacks. Sound signals were controlled in Cool Edit Pro 2.0, which drove an Edirol A/D audio interface and was operated at a sampling rate of 96 kHz, attenuated (PA-5, Tucker Davis Inc., Alachua, FL, USA) and amplified using an amplifier with a flat frequency response of up to 100 kHz (NAD 214, NAD Electronics, Pickering, ON, Canada). Signals were broadcast by two ultrasonic magnetic speakers (MF1-S) with flat frequency characteristics ranging from 1 to more than 40 kHz (Tucker Davis Inc., Alachua, Fl, USA). The spectral composition of both the chirp and the trill at the position of the preparation is shown in Fig. 1b. For more details, see Kostarakos and Römer (2015) and Krobath et al. (2017).

The chirps and trill were broadcast either separately or simultaneously with a signal-to-noise ratio of 0 dB with 75 dB SPL for both signals. The speakers were positioned next to each other along one side of the longitudinal body axis of the preparation at a distance of 15 cm. Sound pressure levels at the preparation were calibrated at the position of the ears using a ½-inch microphone (type 2540, Larson Davis, Depew, NY, USA) connected to a sound level meter (CEL 414, Casella, Bedford, UK). Since the chirp consisted of syllables that increased in amplitude, the sound calibration was performed by presenting a continuous loop of the three last syllables in the chirp.

The tuning of neurons was studied by examining iso-intensity response profiles. We preferred to use iso-intensity responses as they provide tuning information at the suprathreshold level, and, thus, intensity values representing closer communication distances. Tuning was studied using pure-tone sound pulses (duration 50 ms) at frequencies of 1, 2, 5, 10, 15, 20, 30, 40 and 55 kHz, generated with Cool Edit Pro 2.0 at a sampling rate of 192 kHz. All frequencies were broadcast at 80 dB SPL at the position of the preparation, if not stated as otherwise.

### Intracellular recordings

Intracellular recordings were performed from auditory interneurons within the prothoracic ganglion. Animals were anesthetized with chloroethyl, and the antennae, middle and hind legs were removed. The animals were fixed ventral side up on a holder using dental wax. The head was slightly tilted backwards and fixed on the holder, while the tarsi of the forelegs were waxed onto thin wires. The prothoracic ganglion was exposed, and the perineurium was carefully removed above the auditory neuropil, allowing a smooth penetration of the microelectrode. The prothoracic ganglion was covered with insect saline. The preparation was then placed at a distance of 15 cm from the speakers. The ganglion was stabilized between a small, metal platform on its dorsal side and a metal ring on its ventral side, with the platform serving as a reference electrode for intracellular recordings. Microelectrodes were filled with 5% Lucifer yellow CH (Sigma-Aldrich) dissolved in *aqua destillata*, or with 0.8% Alexa 555 and 568 hydrazide (Molecular probes) dissolved in 0.2 M lithium chloride (LiCl) for intracellular staining, using conventional protocols.

Intracellular recordings were mainly performed within the anterior part of the prothoracic ganglion in the area of the auditory neuropil (Römer 1983; Stölting and Stumpner 1998). Neuronal responses were amplified by a BA-01X amplifier (npi electronic) in bridge mode. Fluorescent dyes were iontophoretically injected into the neurons by hyperpolarizing current injection (0.5–5 nA) for 5–30 min. After they were histologically processed and cleared using methyl salicylate, the morphology of the neurons was visualized with a Zeiss Axioplan epifluorescence microscope (Carl Zeiss). Images were taken with an Axiocam ERc 5 s (Carl Zeiss microscopy GmbH, Göttingen, Germany). Neurons were reconstructed manually from image stacks using ImageJ (National Institutes of Health, Bethesda, MD) and Photoshop CS6 (Adobe Systems) software. Neurons were identified on the basis of their morphology and response patterns. Both males and females were used for neurophysiology, as no differences were observed in response properties and structures of the auditory interneurons between sexes.

### Terminology

The terminology used to describe neurons was based on their frequency selectivity and axonal projections. The letters LF indicate low-frequency neurons which are sharply tuned to low frequencies around 2 kHz. The letters BF indicate broad-band neurons which are selective over a bandwidth of more than 10 kHz and respond also to sound frequencies below 10 kHz. The letters HF indicate high frequency neurons that respond strongly to frequencies above 10 kHz and revealed a high sensitivity up to 55 kHz. The third letters indicate axonal projections ascending towards the brain (A), descending towards the mesothoracic ganglion (D), or a T-shaped structure of axons that are both ascending and descending (T). Numbers were added to discriminate between neurons that had similar anatomical features and tuning properties, but differed in their other physiological properties.

### Data recordings and analysis

All recording channels were digitized at 20.8 kHz and a 16-bit amplitude resolution with 0.153 mV per increment, using a CED 1401 micro3 data acquisition interface. Data were recorded to the hard disc of a PC using Spike 2 software (Cambridge Electronic Design). Neural recordings were analysed offline using Neurolab software (Knepper and Hedwig 1997). Responses were analysed by examining peri-stimulus time (PST) histograms.

To determine how strong the response to the chirp was masked by the trill, the neural responses to the trill were averaged over a time window of 300 ms before the onset of the chirp and subtracted from the responses to the chirps presented at the same time as the trill. This difference indicated the degree of neural representation of the masked chirp. In a similar way, the neural response to unmasked chirps was calculated by subtracting spontaneous activity (again averaged over 300 ms before the chirps) from the activity elicited by the chirp. The response to chirps (for both unmasked and masked conditions) was also calculated within a time window of 310 ms after stimulus onset, including 10 ms for the latency of receptor responses. For an individual low-frequency neuron (LFT-1) with a response exceeding the duration of this time window, the window was increased to 500 ms after stimulus onset.

## Results

### Interneurons with no response to the trill

**Fig. 2:**
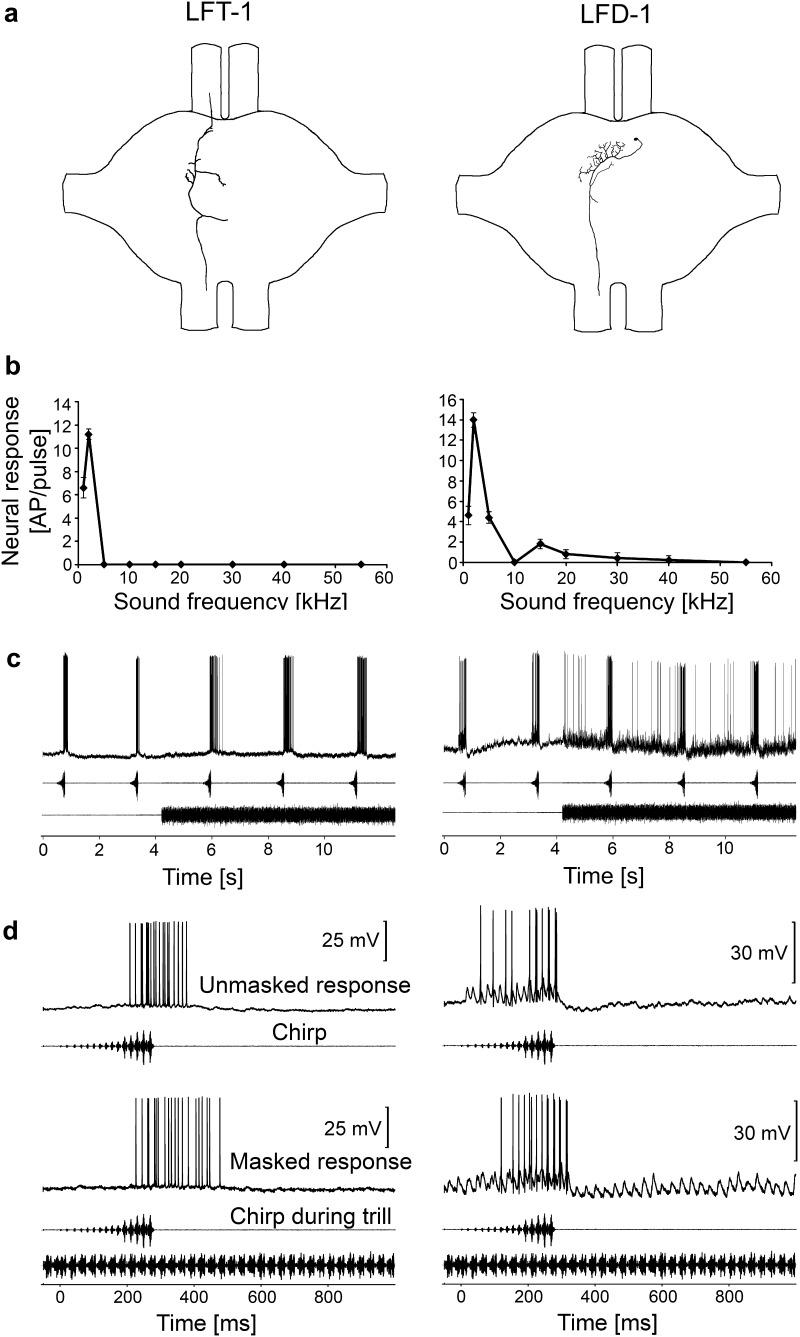
Structure, frequency tuning and response selectivity to chirps of low-frequency interneurons. **a** Reconstructions of intracellular stainings of two types of LFIs in the prothoracic ganglion. **b** Iso-intensity response functions of LFIs at 80 dB SPL [mean ± standard deviation (SD)]. **c** Characteristic examples of LFI responses to chirps presented under unmasking and masked conditions. **d** Characteristic examples of responses of LFIs to a chirp without trill (top) and a masked chirp (bottom)

**Fig. 3:**
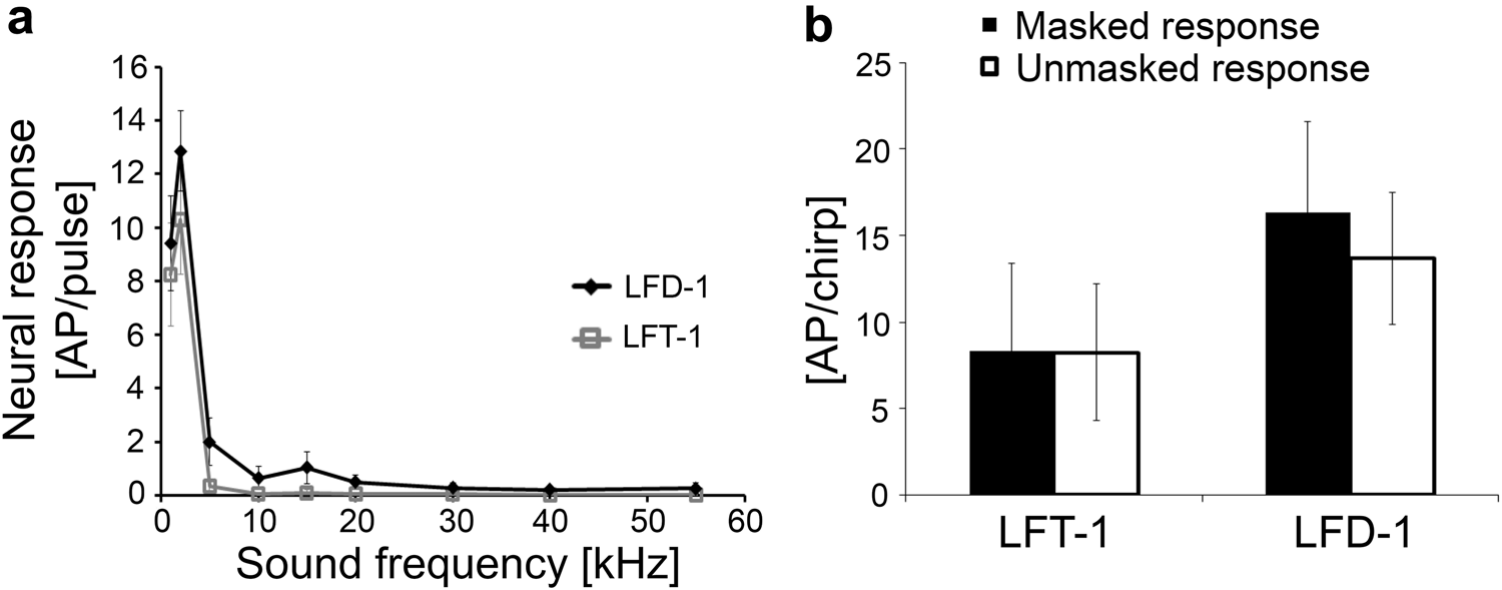
**a** Average of iso-intensity response functions of LFIs at 80 dB SPL [*N* = 4 ± standard error (SE)] for LFT-neurons and *N* = 5 ± SE for LFD-1. **b** Average responses of LFIs to chirps in the unmasked and masked condition (white and black bars, respectively). *N* = 9 ± SD for LFT neurons (two stainings), *N* = 5 ± SD for LFD-1 (three stainings)

We have previously identified two interneurons in the chirper (LFD-1 and LFT) that are narrowly tuned to low frequencies around 2 kHz (Kostarakos and Römer 2015). Since the 2-kHz component was much stronger in the chirper signal than the trill, these two interneurons provided a reliable representation of the chirp even under masking conditions of the trill. These two interneurons also exist in the triller. However, they do not respond to the conspecific trill in this species, but exclusively to the heterospecific chirp, since they are selectively tuned to the low-frequency component of the chirp at 2 kHz, which is strongly reduced in amplitude in the trill (Figs. 2, 3). An analysis of the morphology of LFD-1 revealed dendritic arborisations within the anterior, acoustic neuropil contralateral to the cell body and an axon descending to the mesothoracic ganglion. The cell body is located near the anterior neuropil contralateral to the axon. LFD neurons that have similar morphology and tuning have been described in chirpers (Kostarakos and Römer 2015), indicating their homology. Moreover, the presence of a delay of EPSPs, on the order of 1–2 ms relative to that of auditory afferents, and the overlap of dendritic arborisations with axonal projections of low-frequency receptors in the most anterior part of the neuropil, indicate that they receive direct input from low-frequency auditory receptors. The iso-intensity response curve revealed a sharply tuned response to 2–3 kHz, and almost no response was elicited at frequencies higher than 5 kHz (Fig. 2b).

The LFT revealed similar low-frequency tuning with a response that was exclusively restricted to 1–2 kHz, and, therefore, was highly selective for the heterospecific chirp (Fig. 2b, c). The neuron has an axon that ascends towards the suboesophageal ganglion and an axon that descends towards the mesothoracic ganglion; the cell body is not located within the prothoracic ganglion. Whereas the similarities in the basic structure and physiology of LFD-1 in trillers and chirpers indicate homology, LFT-1 did not exhibit the dense, dendritic arborisations in both hemispheres of the ganglion, as have been described in chirpers (Kostarakos and Römer 2015).

Although similarly tuned, a difference in sensitivity between LFD-1 and LFT was observed. The response of both neurons to the chirp was virtually unaffected by the simultaneous presence of the trill. The response of LFD-1 to the chirp even increased under the masking conditions by an average of 2.7 APs/chirp compared to the unmasked conditions of the chirp (Fig. 2c). The selectivity to the 2 kHz component and the similar response to the chirp and trill is also shown in the average responses of LFIs (Fig. 3).

### Interneurons with responses to the trill and the chirp

**Fig. 4:**
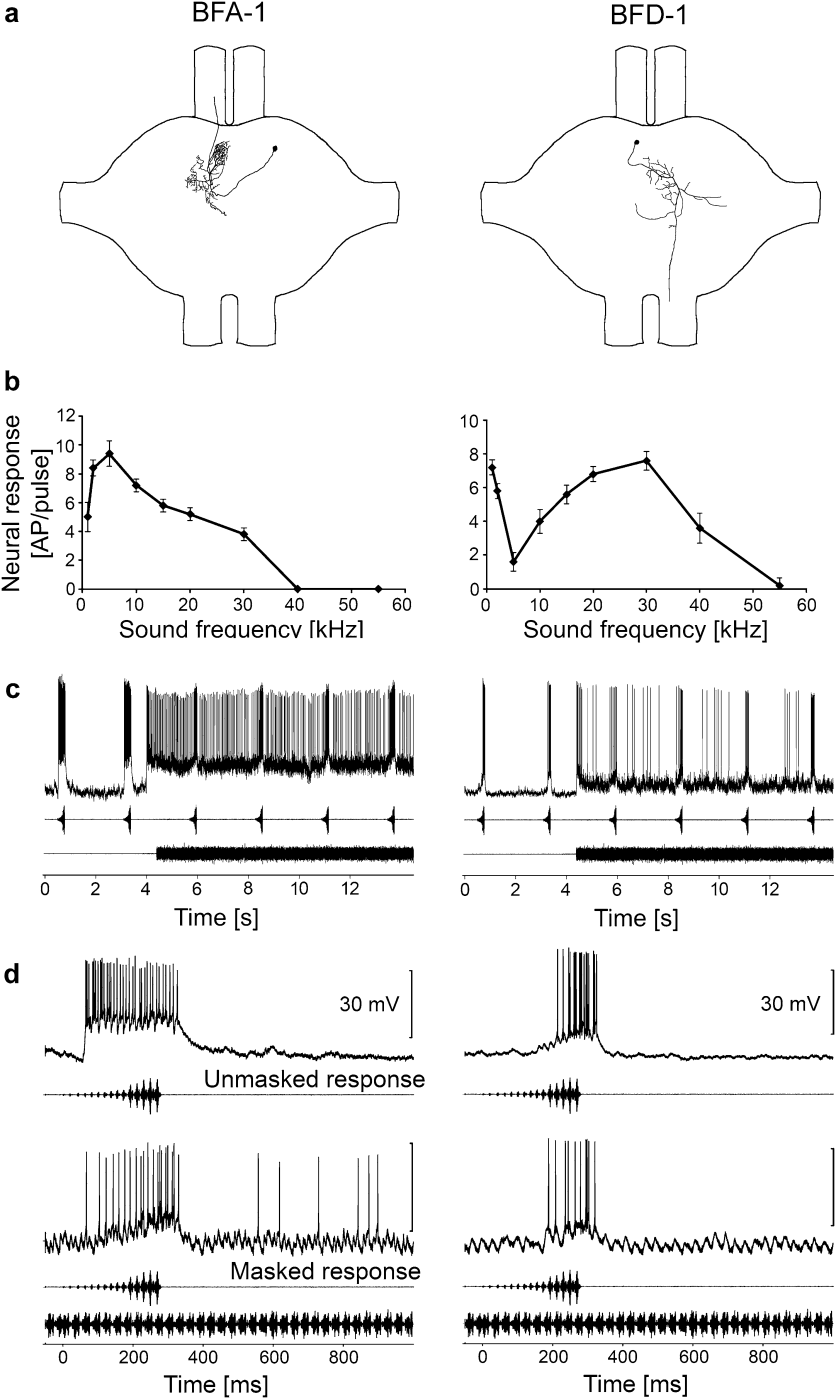
Structure, frequency tuning and response selectivity to chirps of broad-band frequency interneurons, which were also sensitive at low frequencies. **a** Reconstructions of intracellular stainings of 2 types of BFIs in the prothoracic ganglion. **b** Iso-intensity response functions of LFIs at 80 dB SPL (mean ± SD). **c** Characteristic examples of LFI responses to chirps presented under unmasking and masked conditions. **d** Characteristic examples of responses of LFIs to a chirp without trill (top) and a masked chirp (bottom)

**Fig. 5:**
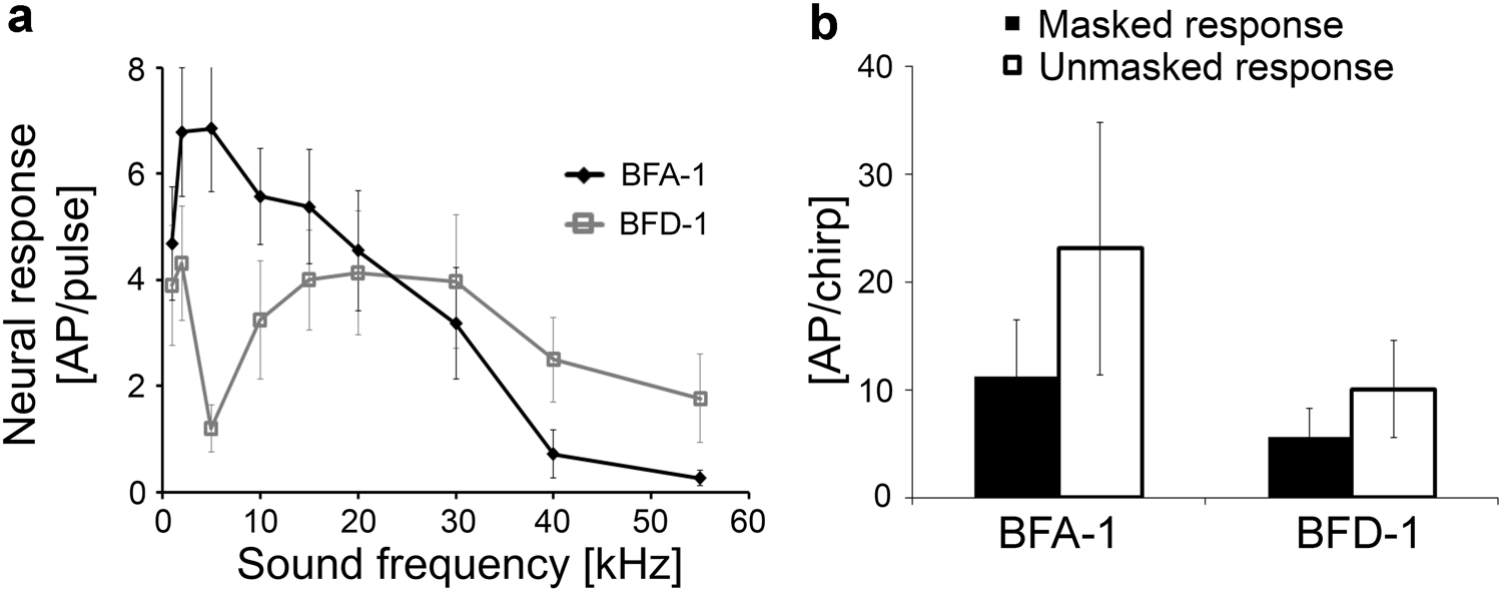
**a** Average of iso-intensity response functions of LFIs at 80 dB SPL (*N* = 8 ± SE for BFA-1 and *N* = 6 ± SE for BFD-1). **b** Average responses of LFIs to chirps in the unmasked and masked condition (white and black bars, respectively). *N* = 9 ± SD for BFA-1 (six stainings), *N* = 6 ± SD for BFD-1 (four stainings)

Unlike LFT and LFD-1 neurons, which have a frequency response restricted to low frequencies around 2 kHz, two interneurons (BFA-1 and BFD-1) are sensitive over a broader frequency range (see iso-intensity functions in Fig. 4b). The response of BFA-1 peaked at 5 kHz, but responses to pure-tone sound pulses could be elicited up to 30 kHz. BFD-1 was sensitive up to 40 kHz. A characteristic feature of this neuron is its selective response to low frequencies, like LFT and LFD-1, whereby a drop of sensitivity was observed at 5 kHz. Both neurons responded to the conspecific trill with ongoing, tonic activity after a pronounced adaptation to the onset of the trill (Fig. 4c). This ongoing activity was higher in BFA-1 than in BFD-1 (Fig. 4c). They also responded to the heterospecific chirp in the unmasked condition, but also to a lesser extent under strong masking conditions of the trill (Fig. 4d). The mechanism resulting in a response to the heterospecific signal has been termed “novelty detection” (Schul et al. 2012), based on the adaptation in response to the masking signal, and the “novelty” of the 2-kHz component in the chirp being not present in the masker. In the chirper, the same response has been described in the homologous interneurons BFA-1 and BFD-1, where it favours the representation of the conspecific signal (Kostarakos and Römer 2015). In both interneurons, the average responses to the chirp were stronger to the unmasked chirp than to the same chirp under masking conditions (Fig. 5b).

The structures of both neurons are shown in Fig. 4a. The cell body of BFA-1 is located close to the dorsal surface of the anterior lobes. An examination revealed the presence of a dense, dendritic branch within the anterior and posterior neuropil contralateral to the cell body. The ascending axon projects contralateral to the cell body towards the suboesophageal ganglion. The cell body of BFD-1 is located near the anterior border of the auditory neuropil, and a descending axon projects towards the mesothoracic ganglion contralateral to the cell body. Dendritic arborisations were seen within the anterior and posterior neuropil. The strong similarities in the structure and physiology of these interneurons in trillers compared to those of chirpers indicate homology.

**Fig. 6:**
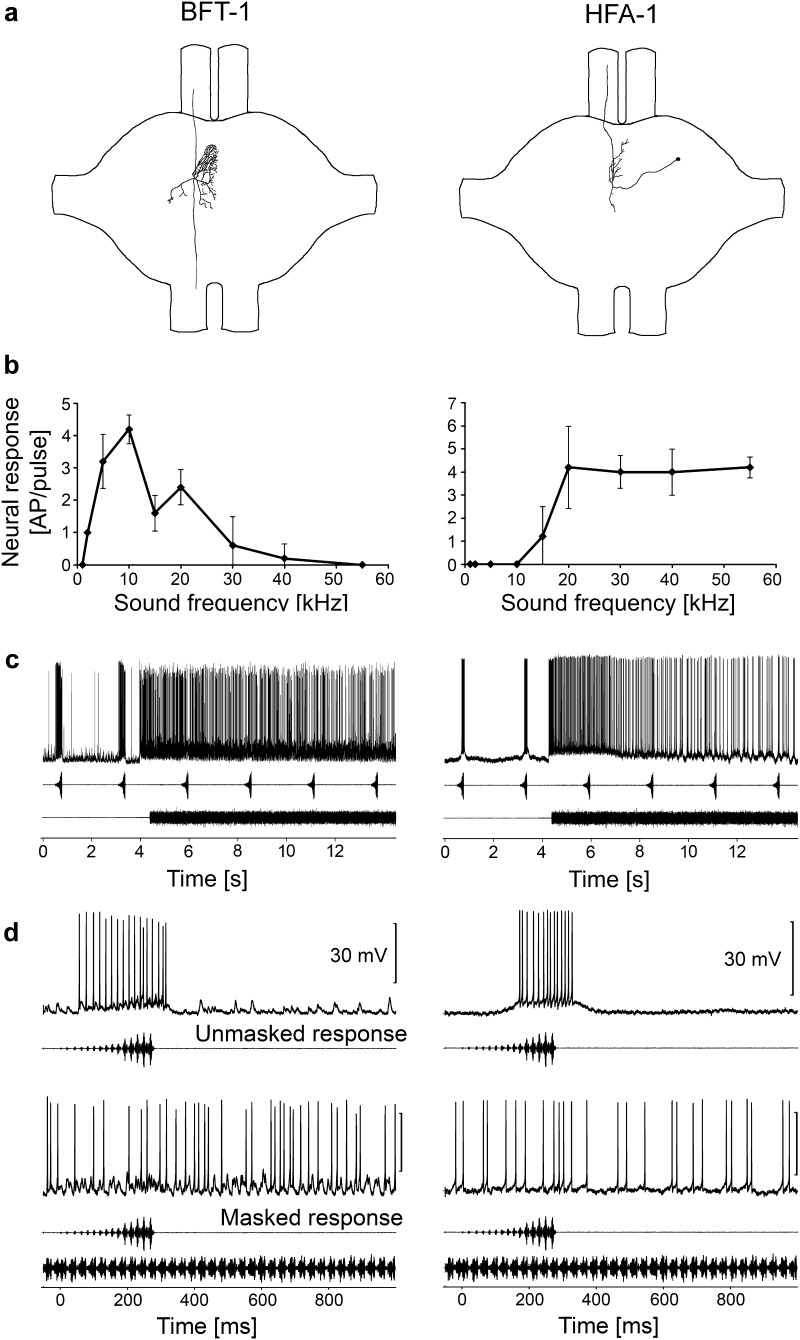
Structure, frequency tuning and response selectivity to chirps of broad-band frequency interneurons not sensitive to low frequencies. **a** Reconstructions of intracellular stainings of 2 types of BFIs and HFIs in the prothoracic ganglion. **b** Iso-intensity response functions of LFIs at 80 dB SPL (mean ± SD). **c** Characteristic examples of LFI responses to chirps presented under unmasking and masked conditions. **d** Characteristic examples of responses of LFIs to a chirp without trill (top) and a masked chirp (bottom)

**Fig. 7:**
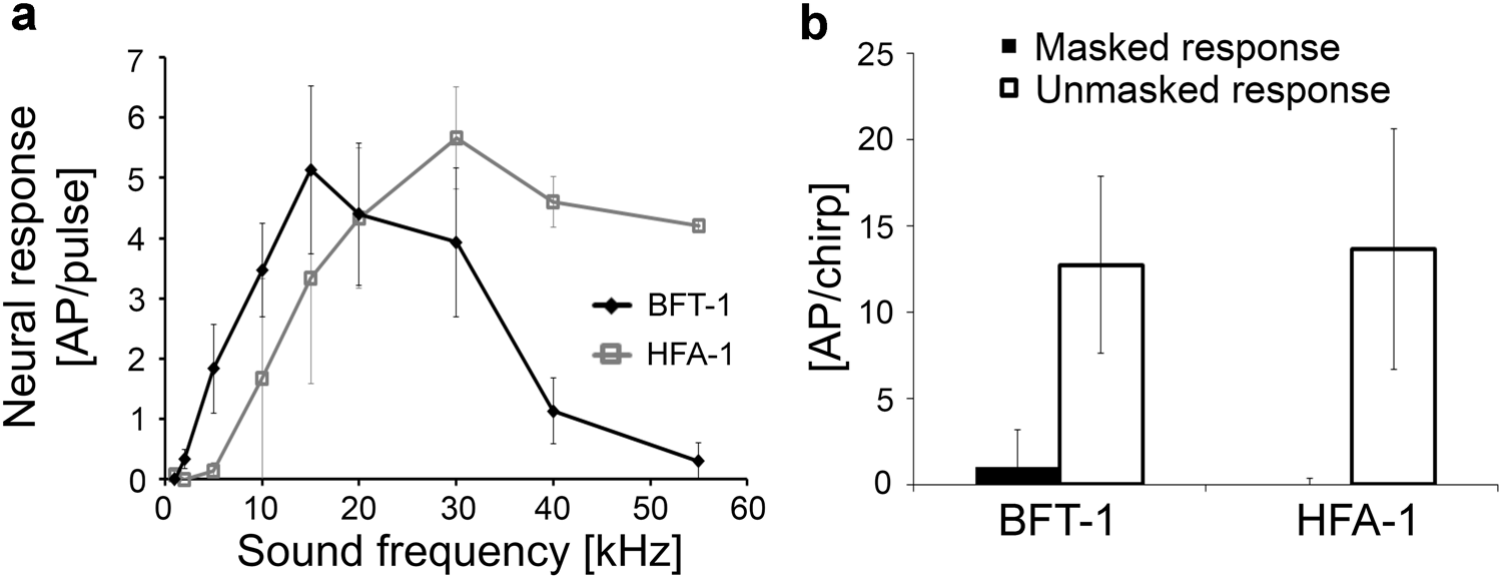
**a** Average of iso-intensity response functions of BFI and HFI at 80 dB SPL (*N* = 6 ± SE for BFT neurons and *N* = 3 ± SE for HFA-1). **b** Average responses of LFI and HFI to chirps in the unmasked and masked condition (white and black bars, respectively). *N* = 6 ± SD for BFT-1 (five stainings), *N* = 3 ± SD for HFA-1 (two stainings)

We also identified two types of interneurons that displayed tonic responses to the trill, but a complete lack of response to the chirp under masking conditions. The iso-intensity functions show that these neurons are broadly tuned. BFT-1 responses peaked at 10 kHz, but its sensitivity decreased at higher frequencies. HFA-1 showed a maximal response in the frequency range from 20 to 55 kHz, but no response was seen at frequencies lower than 15 kHz (Fig. 6b). Due to their sensitivity to high and ultrasonic frequencies, both neurons responded to the chirp and the trill (Fig. 6c, d). The degree of adaptation after the onset of the trill was lower compared to that of the previously described interneurons, allowing a long-lasting, neural representation of the trill. Since the sensitivity to the 2-kHz component of the chirp was small (BFT) or lacking (HFA-1) both neurons responded quite well to the unmasked heterospecific chirp, but not to the chirp under masking conditions. Quantitative values for the response magnitude are summarized in Fig. 7b. The lack of response to the masked chirp in BFT and HFA-1 suggests that the masked response strongly depends on the degree of sensitivity to 2 kHz.

BFT-1 has a descending and ascending axon, and its dendrites branch within the auditory neuropil (Fig. 6a). Its cell body is not located within the prothoracic ganglion. The staining of this neuron revealed a strong similarity to the neuron AN5-AG7 described by Stumpner (1999). The AN5-AG7 neuron exhibited its strongest response in the frequency range of 20–30 kHz matching the best frequency of the calling song. Not all stainings revealed the same dense branching pattern. Since the magnitudes of the response and adaptation were also rather variable, it is unclear whether a variety of BFT neurons exist that share a similar structure to LFT neurons. Due to this uncertainty, we refer to this group as a class of neurons with similar structures and response properties. The structure of HFA-1 is highly similar to the structure of BFA-1, but it is clearly different from BFA-1 due to characteristic differences in tuning (see below). An ascending axon projects into the subesophageal ganglion contralateral to the cell body, which is located close to the anterior lobe of the prothoracic ganglion. Dendritic arborisations branch contralaterally to the cell body. HFA-1 was not presented in our previous manuscript (Kostarakos and Römer 2015), but both its structure and physiology in the chirper was identical to the triller.

**Fig. 8:**
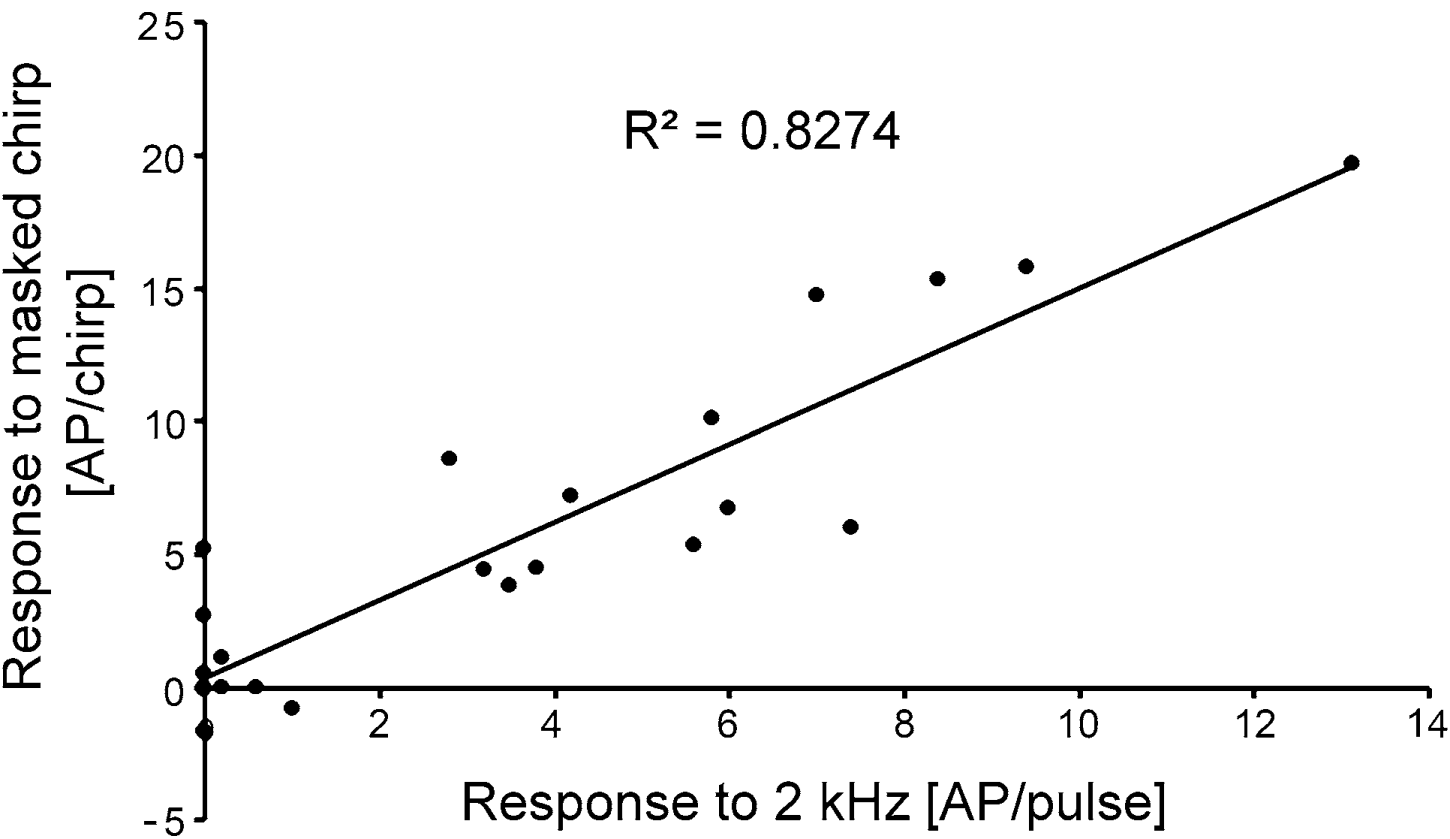
Correlation between the sensitivity to 2 kHz and the neural representation of the chirp under masking conditions for all BFIs and HFIs. Note that the values for the neural representation of the chirp under masking conditions are based on response differences between chirp and trill, therefore, negative values can occur

By including all broad-band frequency neurons (without LFT and LFD-1 with their selective tuning to 2 kHz), a high correlation could be made between the response to the 2-kHz pulse and the response elicited by the chirp under masking conditions (*R*^2^ = 0.8274; Fig. 8).

**Fig. 9:**
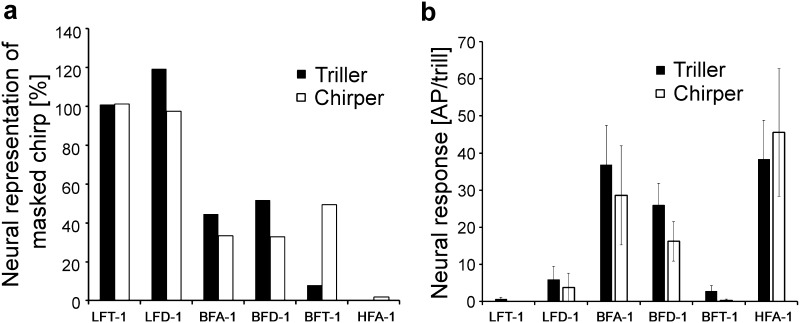
Quantitative comparison of the response magnitude in putatively homologous interneurons of trillers and chirpers. **a** The neural representation of the chirp under masking conditions in percent was calculated by dividing the response to the masked chirp by the response to the unmasked chirp. **b** The neural representation of the trill was calculated over a time period of 2 s, starting 5 s after the onset of the stimulus (mean ± SE.)

Figure 9 illustrates the comparison of the homologous neurons in trillers and chirpers with respect to their responses to the masked chirp and the trill. The response to the heterospecific chirp in the trilling species was either as strong (LFT-1) or even stronger (LFD-1, BFA-1 and BFD-1) than that of the chirper (Fig. 9a). The only exception is BFT-1, where the response to the chirp in the triller was almost completely masked (compare with Fig. 6d), whereas the response was only reduced by 50% compared to the unmasked response in the chirper. This difference between the two species is due to the fact that BFT-1 in chirpers is sensitive to the 2-kHz pulse and shows clear chirp selectivity by novelty detection (Kostarakos and Römer 2015). The quantitative data in Fig. 9b demonstrate that BF- and HF neurons respond sufficiently strong to the trill, allowing trillers to detect their conspecific song.

## Discussion

The aim of the present study was to test the hypothesis that the auditory system of katydids evolved species-specific modifications that allow them to process their own acoustic communication signals. To this end, we compared the tuning and responses of putatively homologous auditory interneurons in a trilling species with those of a previously described chirping species. Like other species using different song types in the *Mecopoda elongata* complex in India, chirper and triller species cannot be discriminated visually or by conventional morphological analysis (Nityananda and Balakrishnan 2006; Dutta 2015; Fig. 1a). The fact that the calling songs of these otherwise morphologically similar species diverge strongly suggests that these songs act as the main premating isolating barrier. This has been confirmed by Dutta (2015) (see also results for the grasshopper genus *Chorthippus*: Vedenina and von Helversen 2003; Gottsberger and Mayer 2007). To function as a hybridization barrier, coding properties of elements in the auditory pathway of female receivers would need to evolve that guarantee the reliable detection and correct interpretation of the signals. We have previously argued that the continuous, loud calling song of the sympatric triller represents a strong environmental constraint, applying selection pressure on the chirper and promoting the evolution of neuronal mechanisms that enable the detection and discrimination of the conspecific chirp under masking conditions (Kostarakos and Römer 2015). Alternatively, we suggested that the described neural mechanisms may be a result of pre-existing properties in the auditory system of Mecopoda.

Regarding the question of homology, we are aware of the fact that we should provide a lineage analysis for each interneuron, tracing each one back to the ganglion mother cell in the developing embryo (Boyan 1993), to comply with the strict definition of the term. However, we support the argument made by Neuhofer et al. (2008) and describe the auditory neurons as putatively homologous in the chirper and the triller *Mecopoda* species based on their similar morphology, as has been done before when comparing auditory interneurons in grasshoppers, crickets, and katydids (Zhantiev and Korsunovskaya 1983; Römer et al. 1988; Stumpner and Helversen 2001; Stumpner and Molina 2006; Stumpner 2002). Our stainings provide strong evidence that LFD-1, BFA-1, BFD-1 and HFA-1 are putatively homologous neurons to their counterparts in chirpers, However, LFT and BFT neurons exhibited stronger variability in their anatomy and physiology. For example, whereas two stained BFT neurons had structures that were almost identical with that of the BFT-1 characterized in chirpers, others had only sparse dendritic arborizations in the auditory neuropil. Thus, for these two interneurons we suggest that they are part of a cluster of interneurons with more variable anatomical and physiological properties.

Our findings regarding the response properties of auditory interneurons in the chirper seem to support our hypothesis of the evolution of adaptive neuronal coding mechanisms: We demonstrated that chirpers use selective tuning and novelty detection as two basic neural mechanisms for signal identification under the masking conditions of the triller. A prerequisite for both mechanisms is either the exclusive (selective tuning) or additional tuning (novelty detection) to a low-frequency component of around 2 kHz that is present in the song of the chirper but much reduced in amplitude in the triller. The presence of both mechanisms sufficiently explains the reliable coding of the chirp even at low signal-to-noise ratios, and these mechanisms have been proposed as evolutionary adaptations in the sensory system of the chirper that enable it to detect signals under masking conditions, namely, during the simultaneous calling of the sympatric trilling *Mecopoda* species (Kostarakos and Römer 2015).

In the current study, however, we were surprised to find interneurons in the triller that had exactly the same mechanisms of selective tuning and novelty detection as in the chirper. Both LFT and LFD-1 interneurons are tuned exclusively to 2 kHz, a frequency component of higher sound energy in the chirper (Fig. 2). Consequently, these neurons respond only to the heterospecific chirper signal. In LFT, the response to the chirp was even enhanced by the continuous trill (Fig. 2c). Similarly, the unusual tuning of BFD-1, with a broad-band sensitivity for high and ultrasonic frequencies and an additional sensitivity peak at 2 kHz, has been described in the same way in the chirper, and we considered the connectivity of this interneuron with auditory receptors as highly adaptive for the mechanism of novelty detection in the chirper (Kostarakos and Römer 2015). Apparently, both chirpers and trillers share interneurons that have a set of conserved coding properties and which have not evolved as adaptations for the coding of conspecific signals. Instead, these features may have been present in a common ancestor. A quantitative comparison of the response magnitude of chirper and triller interneurons also supports this hypothesis (Fig. 9). In interneurons with a sustained tonic response to the trill (BFA-1, HFA-1 and BFD-1), the response magnitude to the conspecific signal in the triller was not higher than that of the chirper.

One possible reason for the failure to demonstrate species-specific adaptations in auditory interneurons of both species could be that both separated only recently, and there was not enough time for such adaptations to evolve. However, a molecular-genetic analysis using a mitochondrial and a nuclear gene revealed that chirpers and trillers separated about 5 million years ago (Stefan Koblmüller, personal communication). It is, therefore, tempting to speculate that the two mechanisms of selective coding and novelty detection in auditory interneurons do not represent adaptations in the chirper that guarantee the detection of the chirp under the masking conditions of the triller song, but instead that the pre-existing properties of the interneurons of the chirpers favoured the evolution of an additional 2-kHz component in their song. In this respect, it would be interesting to compare the spectrum of this song with the spectra of the five song types reported from India (Nityananda and Balakrishnan 2006). They are highly similar at higher and ultrasonic frequencies, but display some differences, especially at lower frequencies.

Selective tuning and novelty detection appear to be common mechanisms used for sensory processing in Ensifera insects, rather than species-specific adaptations to environmental stimuli. For example, selective tuning was described by Schul (1999) as a mechanism for song discrimination in the katydid *Tettigonia viridissima*. The mechanism is based on the tonotopy of the auditory neuropil in the prothoracic ganglion (Römer 1983; Stumpner 1996; Stölting and Stumpner 1998), where the dendrites of interneurons overlap with the axonal arborizations of receptors. The second mechanism of novelty detection has been described in katydids, first, in the context of detection of predatory (bat echolocation) cues under the continuous masking noise of conspecific song (Schul and Sheridan 2006; Schul et al. 2012) and, second, for the *Mecopoda* chirper for conspecific signal detection under the masking noise of heterospecific song (Siegert et al. 2013). Again, the requirements for novelty detection (stimulus-specific adaptation and a spectral difference between masker and relevant stimulus) are common mechanisms of sensory processing that have even been reported for mammals (Nelken and Ulanovsy 2008; Malmierca et al. 2009; Antunes et al. 2010). To maintain responsiveness to the novel stimulus, differences in the spectral properties between the masker and the relevant stimulus must exist, as is the case with the chirper and triller signals. Triblehorn and Schul (2013) described a model of how stimulus-specific adaptation may occur in the dendrites of interneurons for one signal without preventing a response to another signal. By making a comparison with the Indian song types of the *Mecopoda elongata* group, it would be possible to conclude whether the synaptic connectivity with low-frequency receptors represents a property of the common ancestor in all *Mecopoda* species. Currently, we do not know what the selection pressure might have been that would drive such a connectivity (e.g., predator cues).

Whereas the present data and those of Neuhofer et al. (2008) support the hypothesis, that the coding properties of interneurons in the afferent auditory pathway of Orthoptera are evolutionarily conserved and represent plesiomorphic features present already in an ancestor species, two other studies by Stumpner (2002) and Triblehorn and Schul (2009) came to a different conclusion. Stumpner compared the tuning properties of the omega-neuron (ON1) and the AN1 interneuron in four species of closely related Phaneropterine katydids, where *Ancistrura nigrovittata* shows an unusual low-frequency spectrum of the male song (at about 15 kHz) in this duetting species. Tuning of AN1 to this frequency in *A. nigrovittata* is achieved through frequency-dependent inhibition, which is absent in AN1 of the other three species. Thus, a coevolution between song and AN1 tuning is the likely hypothesis, although the reason for the shift of the male song to lower frequencies remains unclear. Since only the selectivity, but not the sensitivity of AN1 to the male song is changed by inhibition, the shift to lower frequencies must have been accompanied with increased background noise, so that frequency filtering was necessary.

Such a selection pressure for frequency filtering does not exist for both Mecopoda species, and the fact that interneurons of trillers exhibit mechanisms that favour the representation of the heterospecific chirp rather than the trill does not mean that the triller is unable to code their own song. BFT and HFA-1 neurons show ongoing tonic activity in response to the continuous trill, and they do not respond at all to frequencies below 10 kHz (HFA-1) or respond only weakly to the 2-kHz component of the chirp (Fig. 6). However, even the interneurons that appeared to be well-adapted in the chirper, allowing it to selectively respond to the chirp (BFA-1 and BFD-1), also respond in the triller with considerable activity to the chirp despite adaptation after the trill onset (Fig. 4c). Likewise, neurons of chirpers tuned to higher frequencies did not reveal any selectivity to the conspecific chirp. Thus, both chirpers and trillers which rely on plesiomorphic features in neuronal processing, reminiscent of a common ancestor of the *Mecopoda elongata* group, are not prevented from processing conspecific acoustic signals.
